# Draft Genome Sequence of Pseudomonas sp. Strain MWU13-2517, Isolated from a Wild Cranberry Bog in Provincetown, MA

**DOI:** 10.1128/mra.00545-22

**Published:** 2022-07-19

**Authors:** Jordan Yaeger, Scott Soby

**Affiliations:** a Biomedical Sciences, College of Graduate Studies, Midwestern University, Glendale, Arizona, USA; b College of Veterinary Medicine, Midwestern University, Glendale, Arizona, USA; University of Arizona

## Abstract

Pseudomonas spp. are dominant in many soils, but little is known about how they interact with other members of the soil microbiome. Pseudomonas sp. strain MWU13-2516, isolated from a wild cranberry bog in Massachusetts, has predicted genes for hemolysins, usually associated with pathogens, and type 6 secretion systems.

## ANNOUNCEMENT

The ecosystems of lower Cape Cod are endangered by sea level rise, erosion, and climate change. These fragile ecosystems are supported by soil microbe interactions, but little is known about how these microbes interact with each other or with the biological or geophysical environment. As part of a larger microbiome project, we sampled bacteria from wild cranberry bogs in the Cape Cod National Seashore to better understand these bacterial populations. Pseudomonas sp. strain MWU13-2517 was isolated from a Pipestone loamy coarse sand soil (https://websoilsurvey.sc.egov.usda.gov/App/HomePage.htm) (1) (42.070624 N, −70.210548 W) by vortexing in sterile water, which was then plated onto King’s medium B (KMB) agar amended with 50 μg mL^−1^ each of cycloheximide and ampicillin, incubated at 26°C for 48 h, colony-purified 3×, and stored at −80°C in 34% glycerol. Isolates from frozen storage were recovered on KMB agar and then grown overnight in KMB broth for genomic DNA (gDNA) isolation using a DNeasy blood and tissue kit (Qiagen). Illumina-compatible gDNA libraries were constructed using a HyperPlus library preparation kit (KK8514; Kapa Biosystems, Roche, USA). DNA was enzymatically sheared to ≈500-bp fragments, end-repaired, and A-tailed. Illumina-compatible adapters with unique indexes (IDT 00989130v2) were ligated to each sample, cleaned using Kapa Biosystems pure beads (KK8002), and amplified with Kapa HiFi enzyme (KK2502). The libraries were analyzed for fragment size using the Agilent TapeStation system and quantified by quantitative PCR (qPCR) with a Kapa library quantification kit (KK4835; Thermo Fisher Scientific; QuantStudio 5). The libraries were then multiplex-pooled and sequenced using an Illumina MiSeq device on a 2 × 250-bp flow cell. The raw reads were assembled using Unicycler v0.4.8 (2) and polished with Pilon v1.23 (3) within the PATRIC Comprehensive Genome Analysis pipeline v3.6.12 (http://patricbrc.org) using default settings, except for the trim setting, which was set to “true” (4). The PATRIC pipeline includes quality control with QUAST (5) and Trim Galore v0.4.0 (https://www.bioinformatics.babraham.ac.uk/projects/trim_galore/) (6) and annotation with RASTtk (7). MWU13-2517 was placed with high confidence within the genus Pseudomonas by a GBDP phylogenetic tree constructed using the Type (Strain) Genome Server (https://tygs.dsmz.de/) (8), but it does not cluster with any named species. Its closest relative was the plant growth-promoting species Pseudomonas palleroniana (digital DNA-DNA hybridization [dDDH_d4_] score = 45.7%).

Pseudomonas sp. MWU13-2517 was assembled into 47 contigs with a genome size of 6,067,066 bp and 60.2% G+C content from 1,375,575 reads and a total of 650,504,622 bases sequenced, providing 107× coverage with an *N*_50_ value of 296,837 bp. The genome contained 5,520 protein-coding, 56 tRNA, and 2 rRNA genes. The presence of predicted genes for hemolysins, which have not been observed in the genus outside the P. aeruginosa and P. syringae groups, and type VI secretion systems may be involved in maintaining their specific niche within the diverse microbiome of the soil (9).

### Data availability.

This whole-genome shotgun project has been deposited at DDBJ/ENA/GenBank under accession number JALMFA000000000, BioProject accession number PRJNA691338, and BioSample accession number SAMN26899419. The version described in this paper is version JALMFA010000000. The SRA accession number is SRR18508977. The RASTtk annotations are available under open license at Zenodo (https://zenodo.org/record/6416124#.Yl3d45PMK3I).
